# Systemic Lupus Erythematosus and Cardiovascular Disease

**DOI:** 10.7759/cureus.22027

**Published:** 2022-02-08

**Authors:** Surajkumar B Jha, Ana P Rivera, Gabriela Vanessa Flores Monar, Hamza Islam, Sri Madhurima Puttagunta, Rabia Islam, Sumana Kundu, Ibrahim Sange

**Affiliations:** 1 Research, Jinan University School of Medicine, Guangzhou, CHN; 2 Research, Universidad Americana (UAM) Facultad de Medicina, Managua, NIC; 3 Research, Universidad Central del Ecuador, Quito, ECU; 4 Research, Faisalabad Medical University, Faisalabad, PAK; 5 Research, Dr. Pinnamaneni Siddhartha Institute of Medical Sciences, Chinoutpalli, IND; 6 Research, R.G. Kar Medical College, Kolkata, IND; 7 Research, California Institute of Behavioral Neurosciences & Psychology, Fairfield, USA; 8 Research, K. J. Somaiya Medical College, Mumbai, IND

**Keywords:** autoimmune heart disease, glucocorticoid-induced cardiomyopathy, pathogenesis of systemic lupus erythematosus, cvd & sle, cardiovascular disease, peripheral arterial diseases, women and heart disease, atherosclerotic cardiovascular disease, systemic lupus erythematosus disease, systemic lupus erythromatosus

## Abstract

Systemic lupus erythematosus (SLE) is a condition in which autoimmune inflammation affects nearly every organ in the human body; it is characterized by a relapsing-remitting pattern. Systemic inflammation and tissue damage can arise from autoantibodies, the creation of immune complexes, and the deposition of autoantibodies, all defined as autoimmune diseases. Women of reproductive age are at a high risk of developing lupus, a chronic systemic condition. Among women between the ages of 15 and 44 years, the female-to-male ratio for the occurrence of lupus is as high as 13:1, while it is only 2:1 in children and in the elderly. In addition to accelerated atherosclerosis, SLE is associated with an increased risk of cardiovascular (CV) events such as coronary artery disease (CAD), peripheral artery disease (PAD), and cerebrovascular accident (CVA). Several SLE-specific processes, including impaired immunological regulation, impaired endothelial cell (EC) function, impaired vascular repair, hyperleptinemia, and traditional risk factors, contribute to early atherosclerosis in the disease. CAD can occur at any stage of the disease's progression, with younger individuals being much more at risk than their age-matched counterparts. This review article aims to provide a unique insight into the relationship between SLE and cardiovascular disease (CVD) by discussing the pathophysiological role of CVD in SLE, outlining screening criteria, and highlighting the treatment options for CVD in connection with SLE.

## Introduction and background

Systemic lupus erythematosus (SLE) is a disease with a relapsing-remitting autoimmune course that may affect practically any organ in the body. It is characterized by the generation of autoantibodies, the formation of immune complexes, and the deposition of autoantibodies, resulting in systemic inflammation and tissue damage [1]. Throughout the Middle Ages, the Latin name lupus (meaning "wolf") was used to describe a variety of disorders that resulted in ulcerous lesions on the lower limbs. The name "lupus érythémateux" was used by French dermatologist Cazenave in the middle of the 18th century. The major turning point in the history of lupus occurred in the early 19th century when an understanding of the difference between cutaneous lupus and lupus vulgaris in the modern sense started to emerge progressively. Early 19th-century work by Kaposi, Sequiera and Balean, and Osler contributed to the discovery of the disease's systemic nature. DNA was later identified as the primary target of antinuclear antibodies, and interferons (IFNs) have played a crucial role in modern research [2]. SLE has a prevalence of 9-241 cases per 100,000 people per year, and an incidence rate of 0.3-23.2 cases per 100,000 people per year, according to research done throughout the world over the previous 15 years [3].

Women of reproductive age have a strong predisposition to develop lupus. Among women between the ages of 15 and 44 years, the female-to-male ratio for the occurrence of lupus is as high as 13:1, while it is only 2:1 in children and in the elderly [4-6]. While it affects people of many races, it is more common among non-Caucasians. SLE is quite rare in Africa while being more common in Europe and the United States, especially among individuals of African origin [7,8]. It is thought that genetic factors interact with environmental exposures throughout the lifespan of an individual to influence susceptibility to develop SLE. The most substantial epidemiologic evidence for the increased risk of SLE is associated with exposure to crystalline silica, current cigarette smoking, use of oral contraceptives, and postmenopausal hormone replacement therapy, while there is an inverse association with alcohol use [9]. New research suggests a link between SLE risk and exposure to solvents, household and agricultural pesticides, heavy metals, and air pollution [9]. Ultraviolet light, vitamin D deficiency, certain infections, and vaccinations have also been hypothesized to be related to SLE risk [9]. Mechanisms that link environmental exposures with SLE include epigenetic modifications, increased oxidative stress, systemic inflammation, inflammatory cytokine upregulation, and hormonal effects [9].

There are many components to the SLE's complex pathogenesis: autoantibodies and immunocomplexes, involvement of the complement system, dysregulation of many cytokines including type I IFNs, and disturbance of the clearance of nucleic acids following cell death are only some of them [10]. Immunomodulators and immunosuppression can alter SLE's natural course [10]. In addition, SLE and treatment-related consequences such as accelerated coronary artery disease (CAD) and higher infection risk contribute significantly to both morbidity and mortality [10]. The 11-50% monozygotic twin concordance and significant risk observed within families reflect a genetic element [11]. Many genes, including HLA, IRF5, ITGAM, STAT4, BLK, and CTLA4, among others, have been associated with a propensity for lupus [11,12]. SLE diagnosis can be difficult, and while numerous categorization criteria have been proposed, their clinical value is still up for debate [13]. The management of SLE is determined based on organ system involvement, and despite the efficacy of various medications in the treatment of SLE, the disease still leads to significant morbidity and mortality among patients [13]. SLE is linked to an increased risk of accelerated atherosclerosis and cardiovascular (CV) events such as CAD, cerebrovascular accident (CVA), and peripheral artery disease (PAD). CV events occur both early and late in the course of the disease, with younger patients being substantially more at risk compared to their age-matched peers. By discussing the pathophysiological role of cardiovascular disease (CVD) in SLE and outlining screening criteria and treatment options for CVD in connection with SLE, this review article aims to provide distinct insight into the correlation between SLE and CVD.

## Review

Pathophysiology

Vascular Endothelium Damage and Cardiovascular Disease in Systemic Lupus Erythematosus

One of the earliest signs of a developing CVD is the malfunction of the endothelial cells (ECs). In most cases, vascular injury is predicted to be accompanied by a faster rate of endothelium healing. The increased atherosclerotic load in SLE patients has been linked to decreased numbers of circulating endothelial progenitor cells (EPCs) and aberrant function of cells involved in vascular repair. As a result, lupus EPCs/myeloid circulating angiogenic cells (CACs) have a reduced ability to develop into mature ECs and produce lower levels of vascular endothelial growth factor (VEGF) and hepatic growth factor (HGF) [14-18]. Taraborelli et al. conducted a case-control study between 2014 and 2016 among a sample population of 40 females (20 cases with 20 matched controls) between the ages of 26-56 years with a disease duration of <5 years [19]. In this study, patients with a history of CVD or risk factors that could impair peripheral artery tonometry were excluded. Enrolled patients were matched to healthy controls with the same exclusion criteria for sex, age, body mass index (BMI), and blood pressure. Patients and controls received a transthoracic Doppler echocardiogram and an evaluation of endothelial function by peripheral artery tonometry. The study concluded that endothelial dysfunction and arterial stiffness are common in people with early lupus who have no known risk factors for CVD. A more extensive study is needed to compare the characteristics associated with the abnormalities [19].

Several soluble adhesion molecules, such as those released following EC injury and considered to be indicators of endothelial dysfunction, are upregulated in SLE and linked to higher coronary calcium scores [20]. Rho et al. conducted a study that evaluated the levels of cytokines like tumor necrosis factor-alpha (TNF-α), interleukin-1 alpha (IL-1α), VEGF, and inflammatory enzymes such as myeloperoxidase (MPO), matrix metalloproteinases (MMP)-9, acute-phase reactants such as erythrocyte sedimentation rate (ESR), C-reactive protein (CRP), and serum amyloid A (SAA), and adhesion molecules like vascular cell adhesion molecule (VCAM), intercellular adhesion molecule (ICAM), and E-selectin in 109 patients with SLE and 78 healthy controls. In this study, in individuals with SLE, the connection between inflammatory indicators and calcified plaque revealed by electron beam CT was investigated. The study concluded that in SLE, concentrations of adhesion molecules and TNF-α are linked to CAD regardless of the Framingham Risk Score [20]. According to Nhek et al., SLE sera can activate platelets, leading to EC activation and proinflammatory mediators in an IL-1-dependent way [21]. In SLE, there is a significant imbalance between EC damage and healing [15]. As a result, people with SLE have defective ECs and poor repair of damaged ECs, encouraging vascular plaque formation.

Dysregulation of the Innate Immune Response

Due to the immensely essential function of type I IFNs in SLE, these cytokines have been studied extensively as a contributing factor to the development of lupus-related CVD. Endothelial function is impaired in lupus patients with a strong type I IFN signature (Table 1) [16]. Lee et al. conducted a study in 70 SLE patients and 31 healthy controls. EPCs in the blood of SLE patients and healthy controls were counted using a colony-forming assay. Real-time polymerase chain reaction (PCR) assessment of the IFN-I-inducible gene MX1 was used to determine serum IFN-I levels. Peripheral artery plethysmography was used to assess endothelial function. When compared to controls, SLE patients had significantly lower levels of EPC colony-forming units [median: 5.7/ml peripheral blood (interquartile range: 1.9-12.8) versus 28.5/ml peripheral blood (14.7-47.3); p=0.0001], and the loss of EPCs was more pronounced in patients with elevated concentrations of IFN-I. These findings support the unique idea that endothelial dysfunction and higher CV risk are connected to EPC depletion produced by elevated IFN-I in SLE [16]. Increased serum IFN activity has been linked to a reduction in endothelial function, although serum levels of high-sensitivity CRP, adhesion molecules, and lupus disease activity have not. This shows that elevated type I IFN signaling may play a key role in increasing CV risk in SLE (Table 1) [22]. Tydén et al. recently showed that in SLE, activation of the type I IFN system could impair endothelial function, even in individuals with mild disease activity. The loss of nitric oxide production and decreased endothelial nitric oxide synthase (eNOS) activity are crucial in developing endothelial dysfunction [23]. According to a study published recently by King et al., type I IFNs have been shown to play a pathogenic function in myocardial infarction (MI) [24]. Ischemic cell death and ingestion of cell debris by macrophages in the heart drive a catastrophic response to MI by activating interferon regulatory factor 3 (IRF3) and type I IFN production via the cyclic GMP-AMP synthase-stimulator of IFN genes [24]. Furthermore, following an MI, animals who were given a type I IFN receptor-neutralizing antibody had their IFN response abated, which improved left ventricular dysfunction and survival [24].

Low-density granulocytes (LDGs), a subset of proinflammatory neutrophils seen in lupus patients, have been postulated to play pathogenic roles in lupus CVD through a variety of mechanisms, including their increased tendency to form neutrophil extracellular traps (NETs) [25]. Carlucci et al. found that SLE patients with overall mild-moderate disease activity have a substantial increase in aortic wall inflammation compared to healthy controls, as measured by 18F-fluorodeoxyglucose (18F-FDG) PET/CT scan. Noncalcified coronary plaque burden (NCB) and endothelial dysfunction were significantly higher in this SLE population [26]. The amount of LDGs was independently correlated with NCB when examining the correlations of lupus-related variables to these vascular abnormalities. Furthermore, an LDG gene profile established by RNA sequencing was linked to high vascular inflammation and high NCB in SLE [26]. An abnormality in neutrophil biology has been linked to an increased risk of early vascular disease in SLE.

Dysregulation of Adaptive Immune Response

In SLE patients, abnormal T cell subsets are a pivotal contributor to endothelial dysfunction and CVD. Tregs are anti-atherosclerotic T cells that suppress atherogenic T cell subsets and inflammation. In SLE, a Treg/Th17 imbalance is frequent, leading to atherosclerosis [27]. Plasmacytoid dendritic cells cause CD4+ CXC chemokine receptor 3 (CXCR3) cells to expand and migrate from the circulation into the artery wall in individuals with SLE, where they may have proatherogenic activities [28]. Baragetti et al. published a five-year prospective study in 2017, which showed that increased numbers of CD4+CC chemokine receptor (CCR)5+ T cells were linked to the development of carotid atherosclerosis in SLE patients (Table 1) [29]. According to these data, increased CD4+CCR5+ T cells in SLE may contribute to atherogenesis [29]. Smith et al. published a study in 2016, which proved that in SLE patients with asymptomatic atherosclerotic plaques, invariant natural killer T (iNKT) cells may produce an atheroprotective effect [30]. Healthy iNKT cells differentiated macrophages toward an anti-inflammatory M2 phenotype in the presence of healthy monocytes and serum from SLE patients with asymptomatic plaque. In contrast, SLE patients with clinical CVD had unresponsive iNKT cells and increased proinflammatory monocytes. Furthermore, the authors found that the anti-inflammatory iNKT cell phenotype was linked to dyslipidemia and was fueled by changes in monocyte phospholipid expression and CD1d-mediated iNKT-monocyte cross-talk [30].

**Figure 1:**
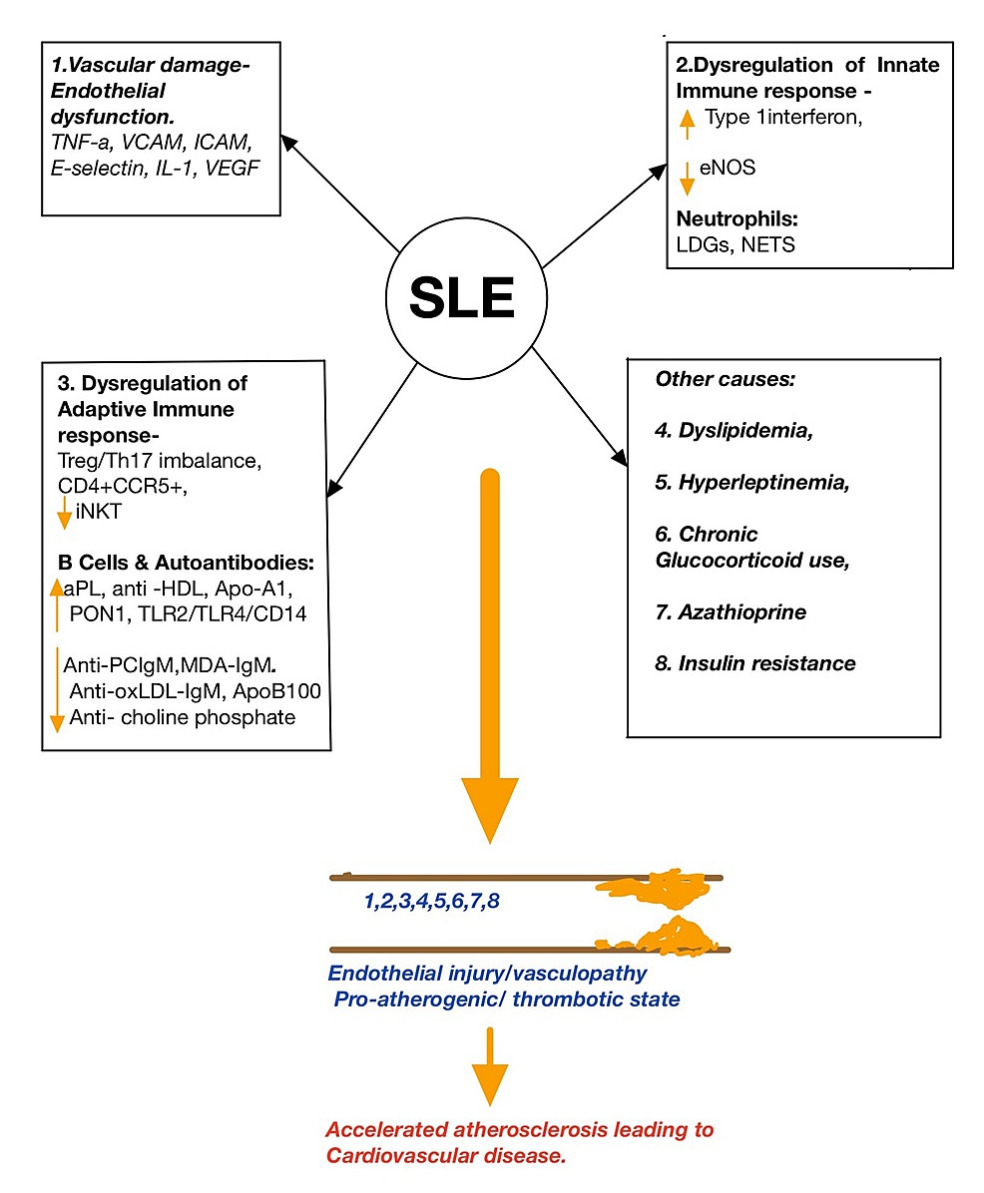
Summary of endothelial damage in SLE leading to CVD SLE: systemic lupus erythematosus; CVD: cardiovascular disease; TNF-α: tumor necrosis factor-alpha; VCAM: vascular cell adhesion molecule; ICAM: intercellular adhesion molecule; IL-1: interleukin-1; VEGF: vascular endothelial growth factor; eNOS: endothelial nitric oxide synthase; LDGs: low-density granulocytes; NETs: neutrophil extracellular traps; Treg: regulatory T cells; Th-17: T helper 17 cells; CD4+: cluster of differentiation 4+; CCR5+: cysteine-cysteine chemokine receptor 5+; iNKT: invariant natural killer T; aPL: antiphospholipid antibodies; anti-HDL: anti-high-density lipoprotein antibodies; Apo-A1: apolipoprotein A-I; PON1: paraoxonase 1; TLR: toll-like receptors; anti-PCIgM: IgM antibodies against phosphorylcholine; MDA: malondialdehyde; anti-oxLDL: anti-oxidized LDL; Apo-B100: apolipoprotein B-100

B cells primarily influence atherosclerosis by producing autoantibodies. B1 cells create harmful IgG antibodies, whereas B2 cells produce beneficial natural IgM and IgA antibodies. Overactive B cells' tendency to produce pathogenic IgG antibodies is also a risk factor for lupus-related atherosclerosis [31]. In particular, antiphospholipid antibodies (aPL) are independent indicators of atherosclerotic plaque development in SLE patients [32,33]. Atherosclerosis develops in SLE individuals with anti-high-density lipoprotein (anti-HDL)-IgG antibodies, allowing low-density lipoprotein (LDL) to enter ECs [34]. According to Kurien et al., anti-oxLDL antibodies are increased by SLE RNP and anti-Ro/LaRNP antibodies [34]. Anti-HDL antibody, anti-ApoA1 antibody, and anti-paraoxonase 1 (PON1) antibody are likely to share a common atherogenic pathway in that they disrupt the balance of PON1/MPO, which increases oxidative lipid modification and interferes with HDL's anti-inflammatory activity, all of which contribute to atherosclerosis [35-37]. Furthermore, anti-ApoA1-IgG induces atherosclerosis in a TLR2/TLR4/CD14-dependent manner through two pathways: it guides the expression of inflammatory factors by activating transcriptional nuclear factor-kappa B (NF-κB); it provides an alternative (or concomitant) signal to phosphoinositide 3-kinase (PI3K) in an Src-dependent pathway, activating L-type Ca2+ channels and potassium/calcium exchangers, resulting in the depolarization of myocardial plasma membrane [38]. Anti-oxLDL-IgM, anti-ApoB100 antibodies, anti-choline phosphate (PC) antibodies, and anti-malondialdehyde (MDA) antibodies are all possible protective autoantibodies in SLE patients. The first three have a synergistic impact in that they lower oxLDL levels, oxLDL uptake, and foam cell formation [39-41]. Anti-PC-IgM boosts Tregs, lowers IL-17 and TNF-alpha in SLE and atherosclerosis, and renders dendritic cells (DCs) immature [42]. Anti-PC-IgM and anti-MDA-IgM used together have a double preventative effect on atherosclerosis [43]. On the other hand, SLE patients were shown to have a low level of protective autoantibodies [41]. By increasing pathogenic autoantibodies and blocking possible preventive autoantibodies, SLE raises CVD risk. Figure 1 presents a summary of endothelial damage in SLE leading to CVD.

Other Causes of CVD in SLE

Dyslipidemia is a predisposing factor for atherosclerosis and a well-established risk factor for CVD. Dyslipidemia is defined as an increase in total cholesterol, LDL, triglycerides, and apolipoprotein B and a decrease in HDL in SLE [44]. Patients with SLE have higher amounts of oxidized and malfunctioning HDL and reduced cholesterol efflux ability and atherosclerosis [45-47]. HDL protects the arteries by boosting the activation of transcription factor 3 (ATF3), which leads to a reduction in toll-like receptor (TLR)-induced inflammatory reactions [48]. In contrast, according to recent research, oxidized lupus HDL enhances proinflammatory responses in macrophages. Indeed, in patients with SLE, HDL stimulates NF-κB, enhances the generation of inflammatory cytokines, and fails to prevent TLR-induced inflammation [49]. The inability of lupus HDL to suppress inflammatory responses is due to a reduced capacity to stimulate ATF3 production and nuclear translocation, which is triggered by oxidized LDL receptor signaling. Indeed, systemic administration of an HDL mimic to lupus-prone animals resulted in considerable ATF3 activation and reductions in proinflammatory cytokine levels, indicating therapeutic potential [49]. In recent studies, impaired cholesterol export capability was strongly linked with vascular inflammation and NCB in multivariate analysis, suggesting that improving HDL function may have considerable cardioprotective implications in SLE [26].

Insulin resistance has been linked to CVD in SLE patients. Recent research has measured insulin sensitivity in SLE patients with a meal-tolerance test. Despite adequate glucose tolerance, intact ß-cell function, and skeletal muscle glucose transporter 4 (GLUT4) translocation, SLE patients had a bi-hormone metabolic anomaly defined by enhanced insulin resistance and hyperglucagonemia. As a result, the authors believe that improving insulin sensitivity may involve more than just addressing insulin resistance [50].

Hyperleptinemia is linked to the progression of atherosclerosis by promoting inflammation through the release of proinflammatory cytokines such as IL-6, TNF-α, IL-17, and other cytokines [51]. In a cross-sectional study published in 2017, Versini et al. stated that there is a lack of data on the link between obesity and SLE. Although studies disagree on the connection between leptin and SLE, a rise in serum leptin levels may enhance systemic inflammation in SLE patients [52]. In contrast, investigations conducted in Egypt in 2018 found that SLE patients have significantly more amount of serum leptin [53].

In general, examining drugs and CVD risk is complex since there might be much confounding by indication. Patients with more severe SLE are more likely to be given glucocorticoids and other immunosuppressive drugs, whereas those with lesser illness may be given hydroxychloroquine alone [54]. Although rising glucocorticoid dosage is closely connected with more significant disease activity, the association between glucocorticoid usage and CVD risk among SLE patients is challenging to understand completely. While glucocorticoids reduce systemic inflammation, which may minimize atherogenesis, their usage is linked to an increase in several traditional risk variables, including total cholesterol, blood glucose, BMI, and systolic blood pressure [54]. More prolonged glucocorticoid usage was found to be independently related to incident CVD events, albeit the relative risk was not assessed in that study [55]. Nikpour et al. also reported that using glucocorticoids doubly increased the risk of MI, angina, and sudden cardiac death [RR: 2.01, 95% confidence interval (CI): 1.19-3.41] [56]. However, these effects have not been repeated in other studies. The difficulty of distinguishing disease activity from glucocorticoid usage, as well as the numerous ways in which glucocorticoid use has been assessed, such as duration of use and maximum, current, or total dosage, may contribute to the disparity among studies [56-59]. Two studies have found that azathioprine usage is an independent risk factor for CVD among SLE patients. One study published by Burgos et al. in 2009 predicted a three-fold higher risk of PAD [60]. And another study published by Haque et al. indicated a three-fold increased risk of MI and angina [61]. Table 1 presents a summary of studies about the pathogenesis of CVD in SLE.

**Table 1:** Summary of studies about the pathogenesis of CVD in SLE SLE: systemic lupus erythematosus; CVD: cardiovascular disease; PCR: polymerase chain reaction; IFN: interferons; FMD: flow-mediated dilatation; CIMT: carotid intima-media thickness; EPC: endothelial progenitor cells: CVRFs: cardiovascular risk factors

Reference	Design	Cases	Controls	Population	Variables	Findings
Somers et al. (2012) [22]	Cohort study	95 SLE patients	38	<55 years, from Michigan Lupus Cohort	Real-time PCR was used to measure serum type I IFN activity. The impact of type I interferons on FMD, CIMT, and cardiac calcification was studied. Patients with CVD were not allowed to participate in the study	Type I IFNs are independently associated with atherosclerosis development in lupus patients without a history of overt CVD
Baragetti et al. (2017) [29]	Prospective study (2012-2017)	40 SLE patients	50 healthy age-matched controls	Mean age: 42 ± 9 years	Clinical history and details on the principal CVRFs were obtained at baseline and after five years. Carotid Doppler ultrasonography was employed to quantify the atherosclerotic burden at baseline and follow-up. The association between basal circulating T cell subsets and atherosclerosis development was evaluated	During the 5-year follow-up, 32% of SLE patients developed carotid atherosclerosis compared to 4% of controls. Increased levels of CD4+CCR5+ T cells were independently associated with the development of carotid atherosclerosis in SLE patients
Lee et al. (2007) [16]		70 SLE patients	31 healthy controls	University of Florida Center for Autoimmune Diseases; 27-45 years, females	EPCs in the blood of SLE patients and healthy controls were counted using a colony-forming assay. A real-time PCR quantified serum IFN-I levels. Endothelial function was determined by peripheral arterial plethysmography	When compared to controls, SLE patients had significantly fewer EPC colony-forming units. The loss of EPCs was more pronounced in patients with high IFN-I levels. In patients with SLE, high IFN-I levels were linked to poor endothelial function

Clinical implications

Evidence of an Increased CVD Risk in SLE Patients

Compared to healthy persons of the same age and gender, patients with SLE have a higher chance of having ischemic heart disease or stroke, referred to as CVD [62]. A population data-based study published by Aviña et al. in 2017, including all British Columbia and other Canadian residents, studied all patients with incident SLE with a healthy group from the general population as a matched cohort. Among 4,863 individuals with SLE (86% female, mean age: 48.9 years), the incidence rates (IRs) of MI, stroke, and CVD were 6.4, 4.4, and 9.9 events per 1,000 person-years, respectively, vs. 2.8, 2.3, and 4.7 events per 1,000 person-years in the comparison cohort. Compared with non-SLE individuals, the fully adjusted multivariable hazard ratios (HRs) among SLE patients were 2.61 (95% CI: 2.12-3.20) for MI, 2.14 (95% CI: 1.64-2.79) for stroke, and 2.28 (95% CI: 1.90-2.73) for CVD. The age, sex, and entry time-matched HRs for MI, stroke, and CVD were highest during the first year after SLE diagnosis: 5.63 (95% CI: 4.02-7.87), 6.47 (95% CI: 4.42-9.47), and 6.28 (95% CI: 4.83-8.17), respectively. Hence, CV events are more common in patients with SLE, especially in the first year after diagnosis. In this patient population, increased surveillance in tracking these potentially fatal outcomes and their modifiable risk factors is necessary (Table 2) [62].

A major international cohort study published in 2006 by Bernatsky et al. involving 9,547 individuals with SLE found 1,255 fatalities, 313 of them caused by CVD (Table 2) [63]. SLE raises the risk of CVD by 50 times in women between the ages of 35 and 44 years. However, the increased risk is more negligible at other ages (7-10 times) (Table 2) [64]. A recent meta-analysis discovered that the prevalence of asymptomatic atherosclerotic disease was higher in SLE patients than controls, with higher carotid intima-media thickness (CIMT) scores and a 2.5-fold increase in the prevalence of carotid plaques, both surrogate markers of subclinical atherosclerosis [65]. Aortic stiffness, a key predictor of early vascular aging measured by aortic pulse wave velocity (aPWV), was shown to be affected to a similar extent in SLE as it is in hypertensive patients, indicating that SLE has a similar impact on early vascular aging [66]. Increased PWV and proximal aorta arterial stiffness were seen in children and adolescents with active SLE, implying that inflammation could factor in the increased arterial stiffness seen in young patients [67]. According to epidemiologic research to date, traditional CVD risk variables, such as hyperlipidemia, cigarette smoking, advanced age, hypertension, male sex, and high CRP, appear to be related to increased CVD risk among SLE patients. However, since previous studies did not look at all of these risk variables in the same populations simultaneously, comparisons of the relative risk associated with each are impossible [68]. According to one study, patients still had a 7-10-fold more significant risk of CVD after controlling for Framingham risk factors [69].

**Table 2:** Summary of studies about the evidence for CVD risk in SLE SLE: systemic lupus erythematosus; CVD: cardiovascular disease; MI: myocardial infarction; IR: incidence rate

Reference	Design	Cases	Controls	Population	Variables	Findings
Bernatsky et al. (2006) [63]	Cohort study (1958-2001)	9,547 cases with SLE	--	>16 years from 7 countries (Canada, US, England, Scotland, Iceland, Sweden, South Korea)	Examined the mortality in SLE patients	1,255 fatalities, 313 of which were caused by CVD
Aviña-Zubieta et al. (2017) [62]	Cohort study (1996-2010)	4,863 cases with SLE (86% females)	10 healthy matched controls per case	Mean age: 48.9 years, from British Columbia and other parts of Canada	IRs of MI, stroke, and CVD were observed and compared	IRs of MI, stroke, and CVD were 6.4, 4.4, and 9.9 events per 1,000 person-years, vs. 2.8, 2.3, and 4.7 events per 1,000 person-years in the comparison cohort
Manzi et al. (1997) [64]	Cohort study (1980-1993)	498 women with SLE	2,208 women of similar age participating in the Framingham Offspring Study	University of Pittsburgh Medical Center	Risk factors associated with CV events occurring in SLE patients were determined	33 first events (11 MI, 10 angina pectoris, and 12 both angina pectoris and MI). Women with lupus in the 35-44-year age group were over 50 times more likely to have an MI vs. the control group

Cardiovascular Disease Screening and Assessment in Systemic Lupus Erythematosus

Recently, Mavrogeni et al. showed that CV MRI could detect silent cardiac illness overlooked by echocardiography. Indeed, 27.5% of SLE patients with regular echocardiograms but silent/past myocarditis, MI, or vasculitis had abnormalities discovered using CV MRI [70]. According to a recent study published by Seguro et al., SLE is linked to increased visceral adipose tissue and altered adiposity distribution [71]. Aortic perivascular adipose tissue density was also linked to aortic calcification in women with SLE, implying that adipose tissue malfunction may play an important role in CVD [72].

In the early stages of acute MI, cardiac troponin T (cTnT) has been postulated to measure myocyte necrosis and damage [73]. In the general population with a low CVD risk, high-sensitivity cTnT has shown promise in predicting CVD [74]. Divard et al. have observed that levels of high-sensitivity cTnT were independently related with subclinical atherosclerosis in asymptomatic SLE patients regarded as low risk for CVD based on conventional risk factors in a recent cross-sectional controlled study [75].

LDGs, as previously stated, have a pathogenic role in lupus CVD [26]. In an uncorrected linear regression analysis, LDG levels were substantially linked with NCB severity and reduced cholesterol efflux capability in SLE. Furthermore, the authors discovered that a neutrophil gene signature was related to vascular pathology in SLE patients. Indeed, some of the genes found to be elevated in high-NCB SLE groups were also shown to be increased in LDGs when compared to normal-density neutrophils. These data indicate that LDG levels in SLE patients might be used to predict CVD risk [26].

Reduced overall antioxidant capacity in SLE patients without typical CV risk factors is linked to subclinical coronary microvascular dysfunction [76]. PON1, an antioxidant enzyme that binds to HDL and protects LDL from oxidation, is lower in those with SLE, and it is linked to vascular damage [77,78]. Anti-PON1 and anti-HDL antibodies were studied as biomarkers for lupus CVD in recent research. It was discovered that anti-HDL antibodies were linked to a greater risk of CVD, while anti-PON1 antibodies were coupled to CIMT in SLE patients [36]. As a result, such antibodies might be used as early indicators for SLE-related premature atherosclerosis [36]. According to a recent study, biomarkers reflecting receptor-activated apoptosis and tissue degradation, such as Fas, TNF receptor 1, TNF-related apoptosis-inducing ligand receptor 2, MMP-1, and MMP-7, are significantly higher in SLE patients with CVD than those without CVD [79].

Management of CVD in SLE

SLE is a distinct CVD risk factor caused by traditional and illness-related risk factors, such as chronic condition activity, lupus nephritis (LN), aPL, and glucocorticoid usage [80]. The use of statins should be based on other conventional risk factors and cholesterol levels. Although the actual risk in people with SLE is overestimated, calculating the 10-year CVD risk using, for example, the systematic coronary risk evaluation (SCORE) is recommended [81]. Blood pressure should be kept at 140/90 mmHg; thus, this should be the general target for SLE patients [82]. Patients with blood pressure higher than 130/80 mmHg who have clinical CVD or a highly predicted CVD risk (>10%) should be treated to reach a goal of <130/80 mmHg [83,84]. SLE was independently associated with a high indicator of in-hospital mortality after percutaneous coronary angioplasty (PCI) and was linked to overall mortality, repeat revascularization, and major adverse CV events in a study based on Taiwan's National Health Insurance Research Database. The study showed the inherent risks of SLE in PCI patients and emphasized the need for better treatment and secondary preventive initiatives for these high-risk individuals [85].

Specific Treatment of SLE

Antimalarial's impact on CV risk in SLE has been analyzed in five studies: one meta-analysis, one cohort study, two case-control studies, and one cross-sectional study. In a meta-analysis, Ruiz-Irastorza et al. showed that hydroxychloroquine is effective in the primary prevention of thrombotic events in patients with SLE and cardiovascular risk factors (CVRFs) [86]. Antimalarials were also linked to a decreased incidence of thrombotic events in individual studies [87-89]. Penn et al. reviewed the medical records of 149 non-diabetic SLE patients, half of whom had received antimalarial therapy [90]. Over 16 years, the researchers measured glucose levels (mg/dL), homeostasis model assessment-estimated insulin resistance (HOMA-IR), and LDL. Compared to the control group, the antimalarial group had reduced fasting glucose levels (87.1 mg/dL against 91.5 mg/dL) and insulin resistance (HOMA-IR: 194 vs. 179). LDL levels were also lower in the treated group compared to the untreated group (LDL of 102 mg/dL vs. 118 mg/dL in the treated and untreated groups, respectively). Ultimately, hydroxychloroquine appears to be beneficial for the primary prevention of thrombotic events in patients with SLE and CVRFs, with a degree of scientific evidence and a grade of recommendation of SIGN 1+ B. Also, with a level of scientific evidence 1 for hydroxychloroquine, it appears to have a favorable effect on glucose levels, insulin resistance, and LDL levels. Still, the degree of SIGN recommendation could not be determined [90].

Limitations

This study does not address the traditional risk factors like smoking, obesity, age, hypertension, diabetes mellitus, menopause, and family history of CAD independently leading to CVD, as we focus specifically on SLE leading to CVD.

## Conclusions

Based on the studies discussed in this article, SLE is linked to an elevated risk of accelerated atherosclerosis and CV events, including CAD, stroke, and vascular disease. CV events can occur both early and late in the course of the illness, with younger patients being substantially more at risk than their age-matched peers. In summary, the clinical implication of this article is to establish a strong link between several SLE-specific processes, including impaired immunological regulation, impaired EC function, impaired vascular repair, hyperleptinemia, and traditional risk factors that contribute to the development of early atherosclerosis in the disease. We believe this article can serve as a tool to overcome these challenges by providing a unique approach to the connection between the two entities, i.e., SLE and CVD, by highlighting the pathogenesis, clinical evidence, screening, and management options. We explicitly delineated the challenges faced by physicians while tackling the various association between SLE and CVD by enumerating the different mechanisms involved, specific treatment-related issues, and management of the disease altogether in this article. Despite considerable advances in understanding the mechanisms of such intricate interactions, more research is needed to evaluate the therapeutic value of treating those components in preventing SLE-related CVD. Also, in spite of the advances in treatment, standardized mortality rates in SLE remain three times higher than in the general population. The risk of mortality is significantly increased due to CVD, which is one of the main factors we covered in this article, others being renal involvement and infections. Continued efforts to perform research on alternative processes that contribute to accelerated CVD in SLE and more studies on screening modalities are required to identify better molecular possibilities, facilitate early diagnosis and primary prevention for therapeutic targeting, as well as an emphasis on secondary preventive initiatives for these high-risk individuals, in the long run, to improve the CV outcomes.
